# High Resolution Full-Aperture ISAR Processing through Modified Doppler History Based Motion Compensation

**DOI:** 10.3390/s17061234

**Published:** 2017-05-28

**Authors:** Jung-Hwan Song, Kee-Woong Lee, Woo-Kyung Lee, Chul-Ho Jung

**Affiliations:** 1 Department of Electronic and Information Engineering, Korea Aerospace University, 76 Hanggongdaehak-ro, Deogyang-gu, Goyang-si, Gyeonggi-do 412-791, Korea; junghwan-song@kau.kr (J.-H.S.); kiwglee@gmail.com (K.-W.L.); 2 CAL Lab., HyperSensing Inc., Yusong-gu, Gwahak-ro, Daejeon 169-84, Korea; chjung@hypersensing.net

**Keywords:** inverse synthetic aperture radar, radar imaging, autofocusing, motion compensation, target recognition

## Abstract

A high resolution inverse synthetic aperture radar (ISAR) technique is presented using modified Doppler history based motion compensation. To this purpose, a novel wideband ISAR system is developed that accommodates parametric processing over extended aperture length. The proposed method is derived from an ISAR-to-SAR approach that makes use of high resolution spotlight SAR and sub-aperture recombination. It is dedicated to wide aperture ISAR imaging and exhibits robust performance against unstable targets having non-linear motions. We demonstrate that the Doppler histories of the full aperture ISAR echoes from disturbed targets are efficiently retrieved with good fitting models. Experiments have been conducted on real aircraft targets and the feasibility of the full aperture ISAR processing is verified through the acquisition of high resolution ISAR imagery.

## 1. Introduction

In modern military and surveillance application, there are increased concerns on the threats from unmanned aerial vehicle (UAV) or small drones intruding into secured areas [1,2]. Naturally demands are growing for accurate target detection and classification through automatic target recognition (ATR). In this regard, image acquisition of remotely flying target is of high concern and high resolution ISAR imaging is one of the promising solution [3,4].

ISAR technique has been widely adopted for target recognition and identification in diverse remote sensing applications. Ideal ISAR processor works on the assumption that the target follows a linear trajectory. Thus, the non-uniform movement of aircraft target becomes a critical error source that should be overcome in ISAR system design [5]. In high resolution ISAR, the difficulty is further aggravated as the acceptable error tolerance should be maintained over long target observation intervals.

In typical radar imaging, the target motion can be separated into translation and rotation directions. Translation compensation consists of range tracking for data alignment in range cells and Doppler tracking for azimuth phase adjustment. Range alignment is relatively simple and discussed in many literatures through envelop correlation [6,7] or global range alignment [8]. Phase adjustment is more complicated and various methods including prominent point processing [9], phase gradient method [10], maximum contrast method [11], minimum entropy method [12] and so on have been actively addressed for SAR and ISAR autofocusing.

On the other hand, the rotational motion induced by the variable angular aspect produces migration through resolution cell (MTRC). Without rotation motion compensation, the processed image would suffer from blurring effects due to target de-focusing. Many of the previously known rotation motion compensation is based on Fourier transformations (FT) [13,14,15]. In general, standard FT based compensation exhibits a good performance for small aspect angle. However, when wide aperture span is employed for high resolution ISAR, blurring effects tend to deteriorate as the rotational motion error is highly sensitive to the target aspect angle. In this case, traditional methods are hardly effective and the quality of the processed images gets easily impaired. Sparse-recovery algorithm has been suggested for azimuth processing of high resolution ISAR [16,17,18,19]. However, its performance is susceptible to the imperfect range tracking of the maneuvering target.

To mitigate this problem, numerous efforts have been made to develop motion compensation (MOCO) techniques that include polar format algorithm (PFA) [10,11], back-projection algorithm (BPA) [20] and joint time-frequency transformation (JTFT) [21,22,23]. PFA, originally developed for medical imaging, is preferred for high resolution SAR in spotlight mode. PFA works on polar-to-rectangular reformation that requires an accurate estimate of angular motion. Thus, its performance is highly vulnerable to the non-uniform errors of the rotational target motion. On the other hand, BPA overcomes the non-uniform motion errors by applying phase filters for all aspect angles. In due course, BPA repeats phase filtering and interpolation for every image pixels that would result in increased computational complexity. JTFT attempts to compensate for the time-varying Doppler shifts by the rotational motions of distributed scatterers and suppress the blurring effect. However, the time-frequency transform decomposes the SAR image frame into multiple time slices in azimuth and as a result, the available aperture length is shortened. Consequently, major target features such as aircraft head or tail parts are obscured or even lost in the process of image slicing and its use for ATR application will be discouraged.

Under expanded observation intervals, targets are more likely engaged in complicated maneuvers and the range trajectories deviate from linear function. The classical range-Doppler algorithm fails in generating well focused images due to the aspect angle variations [24]. As an alternative to conventional non-parametric technique, parametric range-instantaneous-Doppler (RID) imaging has been exploited in recent years [25,26,27,28]. Unlike simple frequency domain operations, RID looks for analytical solution against disturbed target motion errors. The motion disturbance problem is resolved by the parametric estimation of the unknown trajectory. However, the implementation complexity escalates as the aperture length is expanded in high resolution ISAR and the computational burden becomes another major bottleneck. In line with RID, recent research attempts to exploit autofocusing based on Doppler parameters estimation algorithm (DPEA) [29]. Once developed for detection of fast moving target in SAR imagery [30,31], its use for ISAR has been investigated in [32]. DPEA relies on the accuracy of unknown parameters estimation, which is highly demanding as it should maintain a good precision across wide aperture.

In general, there is no optimal solution to the ISAR imaging problem when the target motions are not pre-determined. It is not straightforward to apply the SAR-like parametric approach for ISAR imaging due to the difficulty of estimating non-uniform target motions. In [24], parametric estimation is carried out by modeling the received ISAR echoes as multicomponent quadratic frequency-modulated signals for enhanced ISAR processing. For this purpose, high-order polynomial estimation is investigated through theoretical simulations. However, it is not computationally efficient and its practical application to experimental ISAR data may needs further verification. In this respect, it is a challenging topic to implement an efficient acquisition system for high resolution ISAR imaging that can be applicable to the arbitrarily extended aperture intervals.

In this paper, an efficient high resolution ISAR processing technique is developed that takes full advantage of wide aperture radar echoes. Attempts have been made to develop a general parametric ISAR solution applicable to arbitrary target motions using computationally efficient algorithm. For this purpose, a notion of ISAR-to-SAR processing approach is established that exploits fully extended azimuth bandwidth with robust precision. Both SAR and ISAR make use of the principle that the synthetic aperture is a result of coherent processing of multiple echoes from diverse aspect angles. In this respect, high resolution SAR mode is converted into ISAR scheme through model parameter conversion.

The proposed technique is based on the fundamental ISAR/SAR theory but distinguished from the conventional methods by the unique flow of implementing the SAR-like ISAR processor. Doppler and velocity parameters are independently estimated for the fully extended aperture interval with high precision such that the non-uniformly disturbed target motions are fully compensated and focused at ISAR processor. Conventional JTFT approach is constrained by the limited processing interval. Recent works for Doppler parameter estimation adopt standard SAR mode only and may not be applicable to the full aperture case. To maintain a robust motion compensation over full aperture length is not straightforward and increases computational complexity, as it typically demands iterative parameter estimations.

In this paper, considerable efforts are made to increase the computational efficiency and simultaneously maintain the advantageous performance of the spotlight ISAR. We show that the Doppler history of the wide aperture ISAR signal, after corruption by the severe platform motion, can be fully traced and retrieved with the best fitting model. Afterward, a sub-aperture recombination scheme is employed to accommodate a full aperture ISAR processing such that target features can be effectively preserved across the full observation interval. To verify the proposed algorithm in practical application, an experimental ISAR system is developed. Commercial off-the-shelf (COTS) components are mainly used to maximize the system flexibility. No extra hardware is employed so that the quality of the generated ISAR images mostly relies on the receiver processing capability. Fieldwork campaigns have been conducted on aircraft targets flying in remote distances. Experimental results are analyzed to verify the successful acquisition of high resolution ISAR imagery. Finally, conclusions and further works are discussed.

## 2. ISAR to SAR Approach

**Figure 1:**
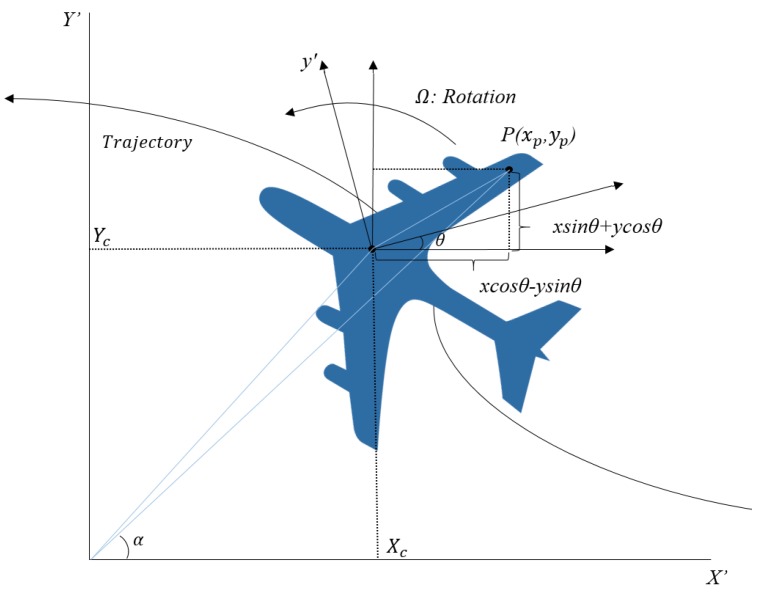
Simplified two-dimensional ISAR geometry.

Conventional ISAR geometry is illustrated in Figure 1. Target position and its three-dimensional motions are projected in two-dimensional image domain. A ground radar is located at the origin of (X’, *Y*’) reference system and the geometric center of the moving target is at ($x_{p},y_{p}$) of (x′,y′) system. Around the target center designated as ($X_{c},\;Y_{c}$) are scattering points arbitrarily located at ($x_{p},y_{p}$) with aspect angle θ and antenna squint angle α. For a distributed target at $P\left(x_{p},y_{p}\right)$, the range function $R_{p}$ is given by [33]:

(1) $$R_{P}\left(t\right)=\sqrt{{\left(X_{c}\left(t\right)+x_{p}cos\theta \left(t\right)-y_{p}sin\theta \left(t\right)\right)}^{2}+{\left(Y_{c}\left(t\right)+y_{p}cos\theta \left(t\right)+x_{p}sin\theta \left(t\right)\right)}^{2}}\;≅\;R\left(t\right)+x_{p}cos\left(\theta \left(t\right)-\alpha \right)-y_{p}sin\left(\theta \left(t\right)-\alpha \right)$$

here θ(t) = $\theta_{o}+\Omega t$, where $\theta_{o}$ is initial rotation angle and Ω is angular rotation rate. Then, the total received signal is:

(2) $$S_{R}\left(f,t\right)=W\left(f,t\right)e^{-j4\pi \frac{f}{c}R\left(t\right)}\int \text{​}\sigma \left(x,y\right)e^{-j4\pi \frac{f}{c}\left[x_{p}cos\theta \left(t\right)-y_{p}sin\theta \left(t\right)\right]}dx\prime dy\prime$$

where W(f,t) is the envelope in the range-Doppler domain and σ is the reflectivity.

When the aspect angle is constrained within a few degrees and the rotation rate is sufficiently low, the polar format grid can be converted into an approximate rectangular type having evenly spaced samples so that conventional FFT can be directly applied. It is typically known in the literature as range-Doppler ISAR [34]. Under such assumptions, the processed point spread function (PSF) is given as:

(3) $$I\left(\tau ,f_{d}\right)=TB\left|sinc\left[B\left(\tau -\frac{2}{c}x\prime \right)\right]\right|\left|sinc\left[T\left(f_{d}-\frac{2f_{o}\Omega }{c}y\prime \right)\right]\right|$$

where τ is the round-trip delay, $f_{d}$ is the Doppler shift frequency, *B* is the signal bandwidth and *T* is the target observation interval. The instantaneous Doppler shift can be expanded as

(4) $$f_{d}\left(t\right)=\frac{2f_{o}}{c}\frac{d}{dt}R_{p}\left(t\right)\;=\frac{2f_{o}}{c}V_{R}\left(t\right)+\frac{2f_{o}}{c}\left[-x_{p}\Omega \sin\left(\theta_{o}+\Omega t-\alpha \right)-y_{p}\Omega \cos\left(\theta_{o}+\Omega t-\alpha \right)\right]\;≅\frac{2f_{o}}{c}V_{R}\left(t\right)+\frac{2f_{o}}{c}\;\left\{\begin{array}{c} -\left[x_{p}sin\left(\theta_{o}-\alpha \right)+y_{p}cos\left(\theta_{o}-\alpha \right)\right]\Omega \\ -\left[x_{p}cos\left(\theta_{o}-\alpha \right)-y_{p}sin\left(\theta_{o}-\alpha \right)\right]\Omega^{2}t \end{array}\right\}$$

When *T* is short, the effective velocity $V_{R}$ can be treated as constant. However, in high resolution ISAR, *T* should be increased in proportion to the required resolution gain. In that case, a simplified approximation of *R*(*t*) and $V_{R}$ may lead to the substantial accumulation of motion errors. Therefore, a refined error estimation and compensation process should be conceived to take into account the extended wide aperture.

### 2.1. Motion Errors in ISAR Doppler History

**Figure 2:**
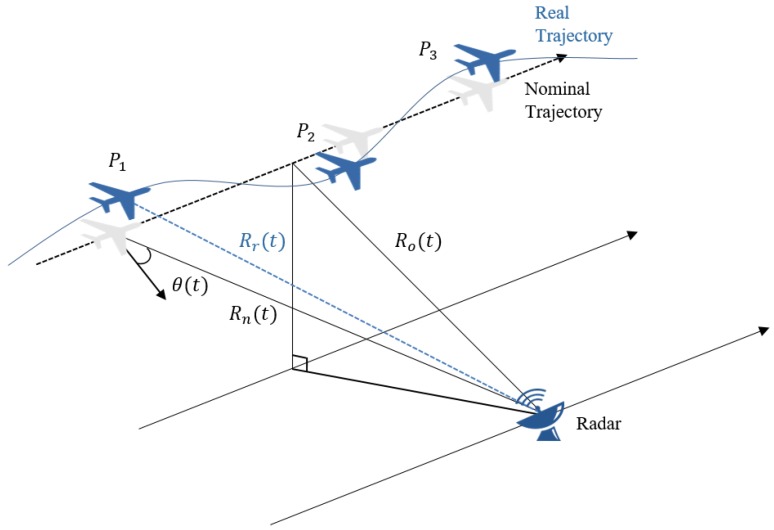
ISAR configuration with non-ideal target motion.

The configuration of typical ISAR operation between radar and a flying target is illustrated in Figure 2. Typically, the real target trajectory $R_{r}\left(t\right)$ deviates from the nominal trajectory $R_{n}\left(t\right)$ and gives rise to undesirable motion errors.

The performance of the acquired ISAR image is mostly dependent on the accuracy of the target range estimation. In a pulsed radar system, stop and go approximation is valid for a narrow antenna beam. Then, the platform velocity is assumed to be nearly constant when the radial velocity error is a fraction of the nominal velocity [35]. Here the range to the target $R_{r}\left(t\right)$ is given by the sum of the nominal range $R_{n}\left(t\right)$ and the translational motion error ΔR(t) as follows:

(5) $$R_{r}\left(t\right)=R_{n}\left(t\right)+\Delta R\left(t\right)\;=\sqrt{R_{o}^{2}+V_{R}^{2}t^{2}}+\Delta R\left(t\right)$$

where *t* is the slow time along the translational direction, $R_{0}$ is the closest range to target and *V_R_* is the target velocity. When the radial velocity and rotational motion errors are sufficiently small in relative to the moving target velocity, the slant range equation can be expanded as:

(6) $$R_{r}\left(t\right)=R_{o}+R_{r}^{\prime }\left(t_{o}\right)\;\left(t-t_{o}\right)+R_{r}^{\prime \prime }\left(t_{o}\right)\frac{{\left(t-t_{o}\right)}^{2}}{2}+\ldots$$

where $t_{0}$ is the reference time at the closest range. Derivative coefficients in the second and third terms correspond to the linear and quadratic range migrations respectively and can be obtained by:

(7) $$R_{r}^{\prime }\left(t\right)\approx \frac{d\Delta R\left(t\right)}{dt}+\frac{V_{R}^{2}t}{R_{o}\left(t\right)}\;≅\;\frac{d\Delta R\left(t\right)}{dt}-V_{R}\sin\left\{\theta \left(t\right)\right\}$$

(8) $$R_{r}^{\prime \prime }\left(t\right)\approx \frac{d^{2}\Delta R\left(t\right)}{dt^{2}}+\frac{V_{R}^{2}\cos^{2}\left\{\theta \left(t\right)\right\}}{2R_{r}\left(t\right)}$$

Here θ(t) is the look angle measured from the reference line of the closest range. Then the range in Equation (6) can be rewritten as follows:

(9) $$R\left(t\right)=R\left(t_{o}\right)+\left\{\frac{d\Delta R\left(t_{o}\right)}{dt}-V_{R}sin\theta \left(t_{0}\right)\right\}\left(t-t_{o}\right)+\left\{\frac{d^{2}\Delta R\left(t_{o}\right)}{dt^{2}}+\;\frac{V_{R}^{2}\cos^{2}\theta \left(t_{o}\right)}{2R\left(t_{o}\right)}\right\}\frac{{\left(t-t_{o}\right)}^{2}}{2}+\ldots$$

Finally, the instantaneous Doppler frequency, after taking into account the translational motion effect designated in Equation (9), is approximated as:

(10) $$\begin{array}{cc} f_{inst}\left(t_{o}\right) & ={-\frac{2}{\lambda }\frac{dR\left(t\right)}{dt}|}_{t=t_{o}}\\ & =-\frac{2}{\lambda }\;\left[-V_{R}sin\theta \left(t_{0}\right)+\frac{V_{R}^{2}\cos^{2}\theta \left(t_{o}\right)}{2R\left(t_{o}\right)}\left(t-t_{o}\right)+\ldots \;\right]\\ & -\frac{2}{\lambda }\left[\frac{d\Delta R\left(t_{o}\right)}{dt}+\frac{d^{2}\Delta R\left(t_{o}\right)}{dt^{2}}\left(t-t_{o}\right)+\ldots \right] \end{array}$$

While the partial sum in the first bracket corresponds to the ideal motion response, the second partial sum represents the Doppler shift error by the disturbed target motion. The unwanted Doppler shift by the non-ideal target motion is separated from the response of the ideal linear trajectory. The disturbance errors are approximated by the hyperbolic power series and corrected later by compensation. For a refined motion compensation, we carry out parameter estimation through polynomial fitting of the range profile curve.

### 2.2. Comparison ISAR with SAR Geometry

**Figure 3:**
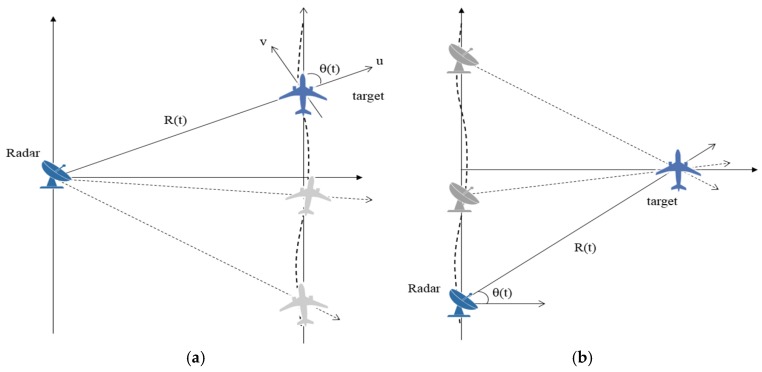
Comparison of ISAR and SAR imaging geometries: (**a**) ISAR case; (**b**) SAR case.

Synthetic aperture is constructed by coherent processing of echoes that come from multi-static target view angles. Figure 3 compares the simplified ISAR and SAR geometrical configurations.

The roles of radar and target are mutually reversed from each side. Basically, they work on the same principle that two-dimensional images are built by the sequential variations of the relative distances between the target and radar in motions. Therefore, Equation (10) is equally applicable to both cases. In this paper, ISAR processing is carried out based on high resolution SAR processing algorithm. The parameters for SAR processing are replaced by the ISAR variables for target tracking and motion compensation. SAR platform velocity and motion errors are converted into the motional variables that describe the ISAR target movement.

Wide Doppler bandwidth is crucial for high resolution SAR imaging. Spotlight SAR mode typically uses antenna steering to collect the wide bandwidth data over extended synthetic aperture length. A refined processing technique is required to accommodate the efficient focusing of the wide Doppler spectrum. In many SAR system, sub-aperture processing is commonly employed to facilitate the long coherent processing interval (CPI) or implement wide-swath mode imaging.

ISAR is distinguished from SAR in that the baseband signal is not pre-determined nor strictly bandlimited. Hence the operation of the high resolution ISAR should be accompanied by the increased pulse repetition frequency (PRF) to avoid aliasing problem. In general, antenna steering is not considered in ISAR and the Doppler property of the echo signal is affected by the observation interval and target movement. Unlike SAR, a full aperture ISAR imaging should be performed against unpredictable baseband signal coming from the uncontrollable target. The difficulty is further aggravated as the aperture length for target observation is limited by the system PRF. In this paper, ISAR full aperture processing is established based on sub-aperture SAR approach such that the wide bandwidth ISAR signal can be efficiently facilitated without necessarily increasing the PRF.

## 3. ISAR-to-SAR Processing with Motion Compensation

In this section, the design of the ISAR-to-SAR processing is discussed. The focusing module is implemented by employing the high-resolution scheme like conventional spotlight. Sub-aperture processing scheme is developed to overcome the lower bound PRF and take a full advantage of wide aperture data.

### 3.1. Motion Compensation Processing

#### 3.1.1. Range Tracking

**Figure 4:**
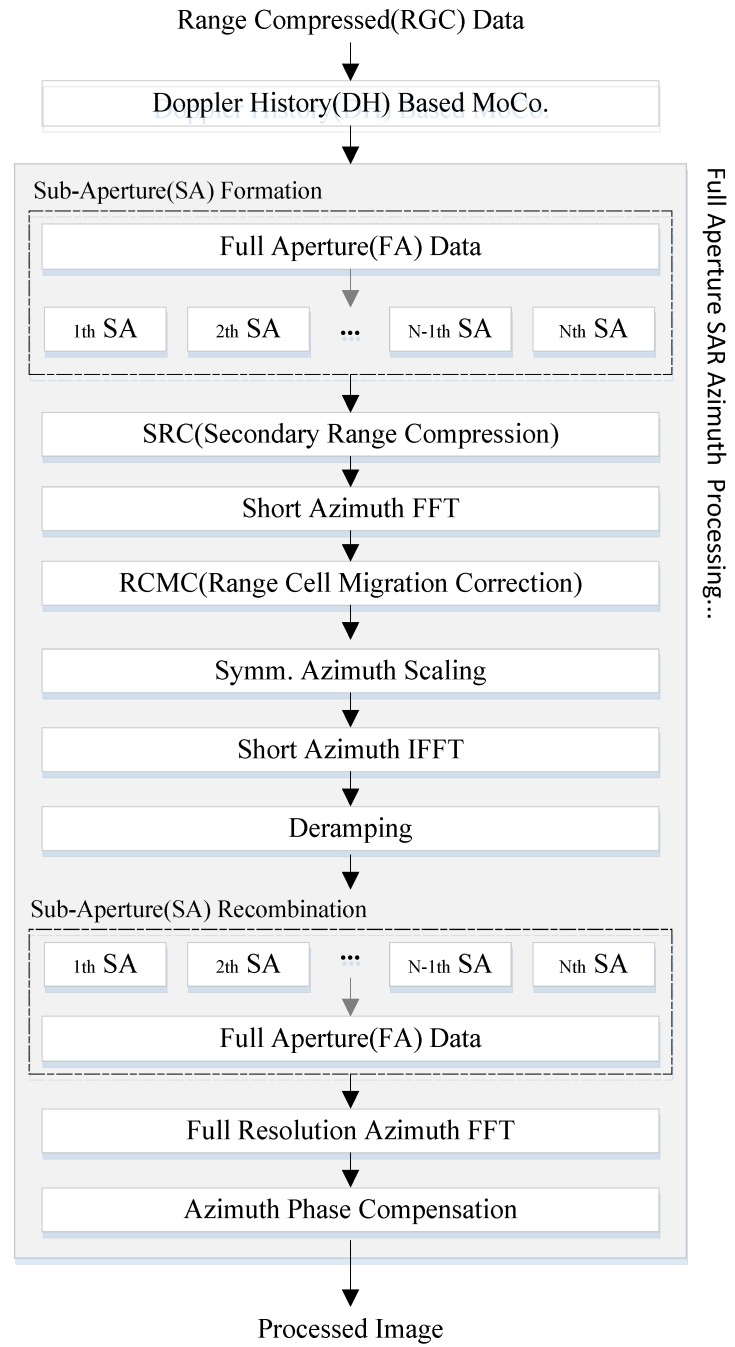
Block diagram of the proposed ISAR full aperture processor with motion compensation.

The proposed ISAR-to-SAR procedure is described in Figure 4. After Doppler history based MOCO is carried out, spectrum analysis (SPECAN) algorithm is applied on the full aperture ISAR data. When combined with a step transform, SPECAN algorithm can be conveniently modified to handle the extended aperture and yield a high-resolution imagery. The established process, denoted as multiple sub-aperture formation and recombination scheme, enables to suppress aliasing effects in Doppler spectrum domain.

Without the prior knowledge of targets movement, the estimation of non-ideal target motion is achieved by engaging a range tracking operation on the received raw data. To reduce data rate and storage capacity, the processing window is limited to pre-determined minimum interval in azimuth. Range compression is conducted to produce range compressed (RGC) data as an input to the peak tracking module.

Non-coherent integration (NCI) is performed in azimuth direction to suppress the noise level. To reconstruct a parabolic SAR geometry model, the range trajectories of prominent scatterers are detected by tracing the peak points. Subsequently, the best estimation of the range trajectory is obtained through random sample consensus (RANSAC) polynomial fitting. RANSAC algorithm is an iterative method to estimate parameters of a mathematical model from a set of multiple observation data. It is known to have robust performance against non-uniform bias errors and generate a good polynomial fitting model. From the constructed range model equation are derived the geometric model parameters such as the slant range of closest approach, reference azimuth time and initial target velocity.

#### 3.1.2. Doppler Centroid Estimation

Doppler centroid estimation is mainly concerned with baseband centroid and ambiguity number. Baseband centroid parameter can be estimated by measuring the matching accuracy of the magnitude or phase distributions. In general, when targets are affected by substantial motion errors, the phase distribution matching is preferred due to the non-uniform variance of the magnitude distribution. Baseband Doppler frequency is calculated by employing the average cross correlation coefficient (ACCC) method. On the assumption that the polynomial range trajectory fitting is well defined, the ambiguity number is directly calculated from geometry model. Then, the absolute Doppler history is retrieved from the estimated range trajectory.

#### 3.1.3. Effective Velocity Estimation

The effective velocity is a major error source and its accuracy directly contributes to the SAR image quality. In ISAR community, iterative autofocusing techniques have been prevalent that utilize maximum contrast [21] or minimum entropy [36]. However, they usually suffer from the increased computational complexity and processing time. In this paper, an effort is made to implement a hybrid scheme to reduce the computational cost. At first the initial velocity is estimated from the predicted geometry model. A coarse estimation is followed using the look mis-registration (LMR) method [29] that has the advantage of fast convergence rate. Then, the maximum contrast processing is conducted such that precise velocity estimation is evaluated within a relatively short time.

#### 3.1.4. Motion Compensation Based on Doppler History

Non-uniform target motion is modelled by *n*th order polynomial. For this purpose, RANSAC fitting is applied to the Doppler history curve and the corresponding polynomial fitting series is expanded as:

(11) $$f_{inst}\left(t\right)=c_{o}+c_{1}t+c_{2}t^{2}\ldots =\;\overset{\infty }{\underset{n=0}{\sum }}c_{n}t^{n}$$

It is simplified into finite terms while the highest degree, denoted in terms of motion index, determines the matching accuracy. The estimated trajectory having $N^{th}$ motion index is given by:

(12) $$R\left(t\right)=\;\sum_{n=0}^{N+1}a_{n}t^{n}$$

The choice of motion index is made by the threshold level designating the inlier samples of RANSAC fitting. After repeated simulations, we choose 5 Hz as an appropriate threshold level. Higher order motion index reflects the increased non-linear target motions from. In due course, range migrations and phase shifts are induced by high order motion effects and should be compensated for. The complexity of the compensation is accelerated in proportion to the observed full-aperture length.

The polynomial coefficients of Equation (12) are obtained from the estimated trajectory model. They are calculated using the relationship of the Doppler frequency and range profile given as $f_{inst\left(t\right)}=\frac{2}{\lambda }\frac{dR\left(t\right)}{dt}$, which leads to:

(13) $$a_{n}=\left(-\frac{\lambda }{2}\right)\left(\frac{1}{n}\right)c_{n-1}$$

When the target trajectory deviates from the parabolic curve, the approximated motion errors are represented as a finite polynomial of:

(14) $$\Delta R\left(t\right)=\;\overset{N+1}{\underset{n=3}{\sum }}a_{n}t^{n}$$

After the polynomial coefficient estimation is completed, the time delay error Δτ and the corresponding phase compensation term Δϕ are given as:

(15) $$\Delta \tau \left(t\right)=\frac{2\Delta R\left(t\right)}{c}\;\Delta \phi \left(t\right)=\exp\left\{\frac{4\pi f_{o}\Delta R\left(t\right)}{c}\right\}$$

The calculated errors in Equation (15) are applied as the compensation terms for the autofocusing process.

### 3.2. ISAR to SAR Full Aperture Processing

As the geometries of ISAR and SAR are conceptually identical, the full aperture SAR processing is naturally applicable to wide-aperture ISAR data. The proposed ISAR-to-SAR technique attempts to convert conventional spotlight SAR algorithm to wide aperture ISAR data. They share many common features while the sub-processing modules are applied in reversed order for each case. As described in Figure 4, the full aperture processing is carried out after MOCO is completed.

For range compression, the range Doppler algorithm (RDA) has been employed but it can be replaced by other SAR processing algorithm such as frequency scaling algorithm (FSA) [33], chirp scaling algorithm(CSA) [37], and Omega-K algorithm [34]. The full aperture azimuth processing consists of sub-aperture division and full aperture retrieval. For computational efficiency, SPECAN algorithm is employed for the rapid azimuth processing of each sub-aperture.

In sub-aperture formation, the range compressed data is divided into multiple blocks in azimuth direction. The number of sub-aperture group is chosen such that the partial Doppler bandwidth of each sub-aperture should not exceed the PRF. A finite overlap is allowed between adjacent sub-apertures to minimize erroneous mis-alignment in the process of recombination. In this paper, the maximum overlap margin is set as 3% of the sub-aperture bandwidth.

Azimuth scaling is useful for enhanced processing accuracy and employed for SPECAN algorithm. After sub-aperture formation, the azimuth focusing is carried out and followed by the secondary range compression (SRC), short azimuth FFT and range cell migration correction (RCMC). Symmetric azimuth scaling is performed to compensate for the range dependent phase history and correct the geometric distortion. It also helps to suppress the errors from the level imbalances of antenna beam pattern and non-zero squint angle. After phase history alignment, deramping operations are repeated for the azimuth focusing of each sub-aperture interval. Finally, the processed blocks are recombined through sub-aperture recombination to form a full aperture and transformed to image domain following the full resolution azimuth FFT.

## 4. System Implementation and Data Acquisition

### 4.1. System Implementation

**Figure 5:**
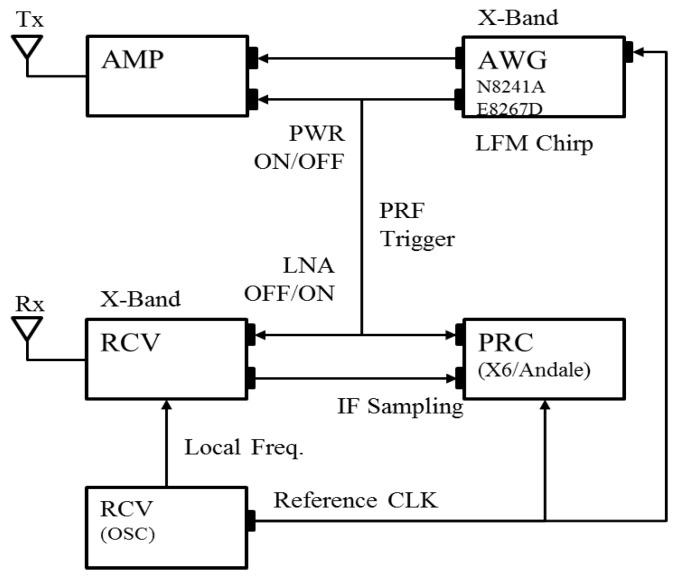
Block diagram of developed ISAR system.

To evaluate the proposed algorithm in real implementation, an experimental ISAR system is developed. The system block diagram is shown in Figure 5 with a brief illustration of signal connections and control flows. To maximize the system flexibility, commercially available off-the-shelf components are mostly employed so that we can easily adjust the operating parameters such as pulse width, signal bandwidth and PRF. Depending on the range, velocity and size of the target, the processing gain is varied by adjusting the time-bandwidth product and the system PRF.

Table 1 summarizes the general specifications of the developed ISAR system. Extra efforts have been made on RF power amplifier and the low noise receiver to compensate for the low output level of AWG. The transmitter operates at X-band of 9.66 GHz. The azimuth processing gain is set around 30 dB at default.

**Figure 6:**
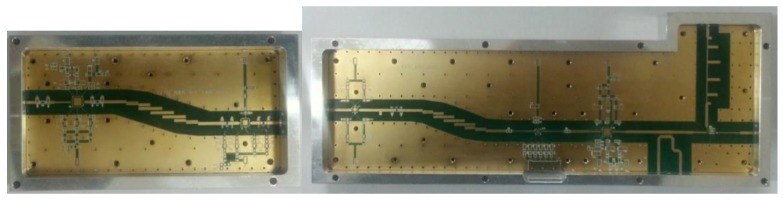
AMP (left) and RCV (right) modules.

The developed amplifier (AMP) module provides an output level up to 40 dBm and the maximum detection range can be extended to several kilometres. RF receiver (RCV) consists of low noise amplifier (LNA) and local oscillator. It is controlled by ON/OFF trigger signal to avoid intrusion of the amplified noise during the receiving window time. Figure 6 shows the implemented AMP and RCV modules.

An arbitrary waveform generator (N8241A) is used for the generation of baseband I/Q signal. The generated baseband signal is up-converted by vector signal generator (E8267D). The bandwidth of the transmitted linear frequency modulation (LFM) is extended up to 500 MHz to enable high resolution imaging of sub-meter level. AWG also functions as a main controller that provides stable trigger signals for the ON/OFF control of AMP and RCV modules.

**Figure 7:**
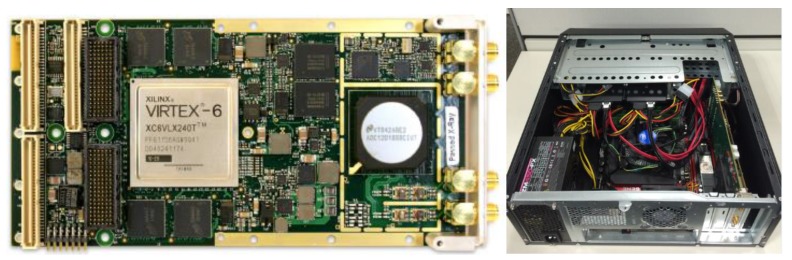
PRC module and data recorder box.

Intermediate frequency (IF) sampling is adopted to minimize the I/Q imbalance problem. Digital down conversion (DDC) scheme is used to receive I/Q signals directly from RCV. For this purpose, PRC module is developed using the X6-GSPS from Innovative Integration inc. that provides high-speed A/D sampling at 12-bit resolution and 3.6 gigasamples per second (GSPS). To accommodate the massive data capacity, multiple solid-state drive (SSD) recorders are connected through PCI express interface that provides a transfer rate of 2 GB/s. Figure 7 shows the PRC and data recorder modules. The control signal for PRC module is relayed from AWG.

### 4.2. Experimental Field Work and Data Acquisition

**Figure 8:**
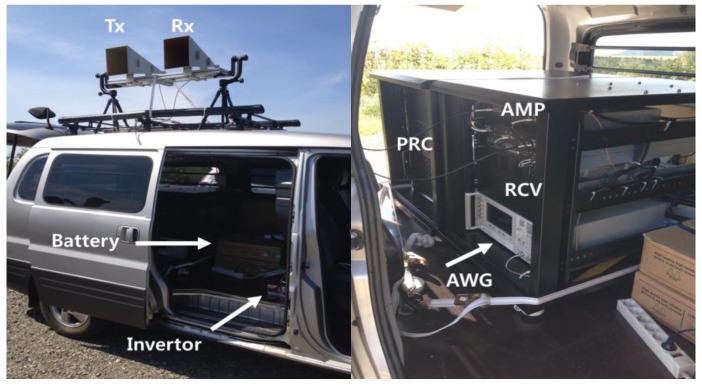
Test equipment on-board a commercial vehicle for ISAR experiment.

Figure 8 illustrates the test equipment setup for the field campaign of ISAR data acquisition. The developed ISAR system is installed on-board a commercial vehicle and connected to the additional power module. The power supply is drawn from the vehicle battery by way of an invertor. Transmit and receive antennas are mounted on the vehicle roof. Standard high-gain horn antennas are chosen to reduce weight burden and minimize internal leakage. Antenna elevation is manually adjusted toward the flying target.

Wideband LFM chirp pulses specified in Table 1 are transmitted at PRF of 800 Hz. Initially, experiments are conducted against a large jumbo-jet which has a relatively stable trajectory. Then, for a comparison, the procedure is repeated against a smaller aircraft to investigate the performance in harsh environment. In our experiment, Boeing 747 and Cessna 208 aircrafts are chosen as they have distinguished cruising stabilities and motion behaviors. The experimental parameters of ISAR data acquisitions are summarized in Table 2. The distances to the targets and their exposure durations are similar in both cases. However, the Doppler bandwidth of the echo signal from Boeing 747 is far higher than the Cessna case. This is attributed to the difference of their flying velocities and corresponding full-aperture lengths.

Having a long aperture length is beneficial to the improved resolution but it is a highly demanding task to maintain a consistent accuracy throughout the whole aperture interval. A good ISAR system should cope with the unpredictable target responses regardless of the aperture length. Accordingly, the full aperture algorithm proposed in this paper is evaluated against two distinguished targets.

## 5. ISAR Experiments and Processing Results

The performance of the proposed technique is evaluated through field experiments. The Doppler histories of Boeing 747 and Cessna 208 are analyzed and processed through the sequences in Figure 4. The distinguished properties of the generated ISAR images are compared to assess the flexible use of the ISAR-to-SAR approach.

### 5.1. Processing Results of Boeing 747

**Figure 9:**
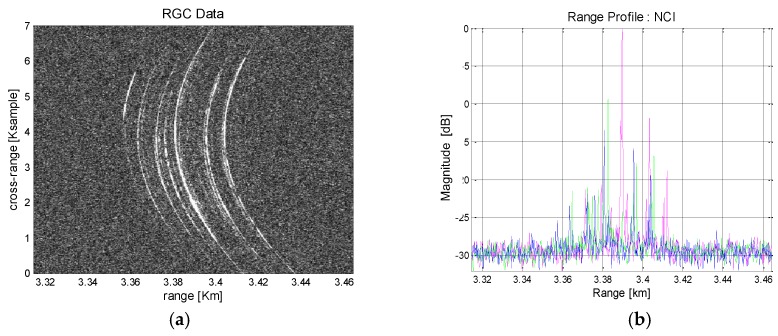
Boeing 747 ISAR data after range compression: (**a**) Range compressed image; (**b**) Integrated range profiles.

Boeing 747 flies with high velocity and requires a relatively wide aperture. Figure 9 shows its RGC pattern and the corresponding range profiles in azimuth direction. The signatures of prominent scatterers are well observed in Figure 9a. They are widely distributed across the long aperture with high curvature and a simple range tracking approach is not easily applicable. NCI across the azimuth is employed to increase SNR so that peak detection and range tracking is facilitated. The integrated range profile is shown in Figure 9b. Signatures from multiple scatterers are distinguished by colors. The strong responses are likely to have come from the aircraft body and engines.

**Figure 10:**
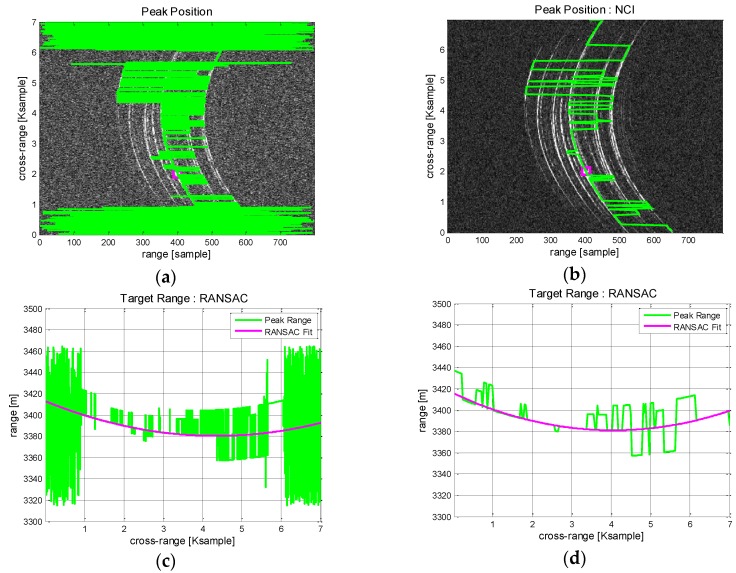
Boeing 747 ISAR data range tracking results: (**a**) Peak detection; (**b**) Peak detection with NCI; (**c**) Trajectory modelling by RANSAC; (**d**) Trajectory modelling by RANSAC with NCI.

In Figure 10, the range trajectory is estimated through RANSAC polynomial fitting. For comparison, the process is performed without and with NCI. Without NCI, there appear considerable bias errors in peak detection (Figure 10a) and the subsequent range tracking suffer from unstable fitting accuracy. These undesirable errors are mostly removed after NCI applied (Figure 10b) and the peak responses can be traced with high precision. Then, RANSAC polynomial fitting is applied to estimate the range trajectory. Figure 10c shows the result that present a moderate range tracking performance hindered by continuous bias errors. Figure 10d, obtained with NCI, exhibits robust tracking performance and a good modelling of the range trajectory. As predicted, the case with NCI exhibits a better performance and should be a favored choice.

**Figure 11:**
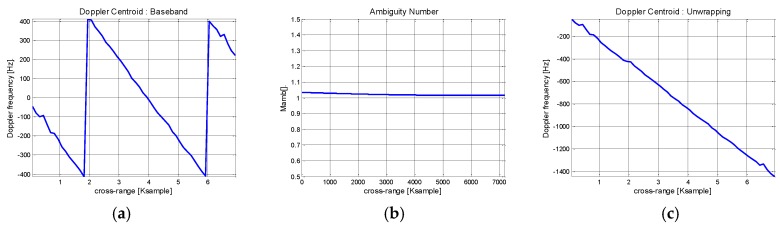
Doppler centroid estimation of Boeing 747 ISAR data: (**a**) ACCC based Doppler history estimation; (**b**) Ambiguity number estimation; (**c**) After phase unwrapping.

Doppler centroid estimation is carried out from the previously obtained range information. For this purpose, the ACCC algorithm is applied across the azimuth and the results are shown in Figure 11. Initially, aliasing effects are visible along the calculated Doppler history in Figure 11a. On the assumption of parabolic range trajectory, the ambiguity number can be directly estimated from the geometry model and Figure 11b shows the result. To discard the ambiguity, phase unwrapping is applied and the absolute Doppler history is restored in Figure 11c.

**Figure 12:**
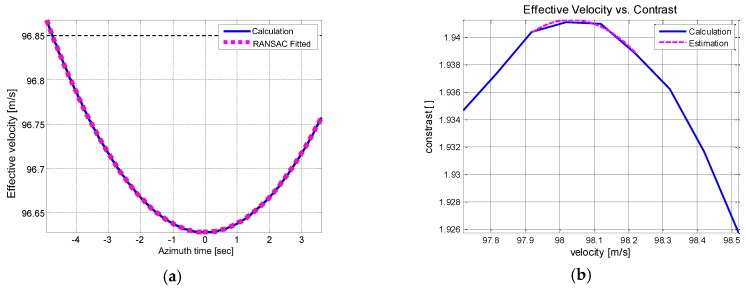
Effective velocity estimation of Boeing 747 ISAR data (**a**) Initial coarse velocity estimation (**b**) CMA based effective velocity estimation.

Target velocity can be derived from the range trajectory and Doppler centroid. Initially, the velocity is calculated from the range trajectory. Then the best fitting model is coarsely estimated through RANSAC fitting. The results are compared in Figure 12a.There are found disturbance errors across the azimuth time that should be corrected to enhance the image quality. To elaborate the evaluation and attain a refined result, autofocusing scheme is applied to the coarse velocity estimation.

The contrast maximization autofocus (CMA) is implemented across the full azimuth interval to search for the best estimation of the effective velocity. Given the fixed velocity, the image contrast is defined as the ratio of the image spatial variance to the spatial mean of the target area and gives a measure of image focusing. The measured image contrast is plotted against velocity in Figure 12b. The contrast level is maximized at 98.03 m/s, which is taken as the best estimation of the velocity. This method is computationally efficient and conveniently applicable to any targets having prominent scatterers.

**Figure 13:**
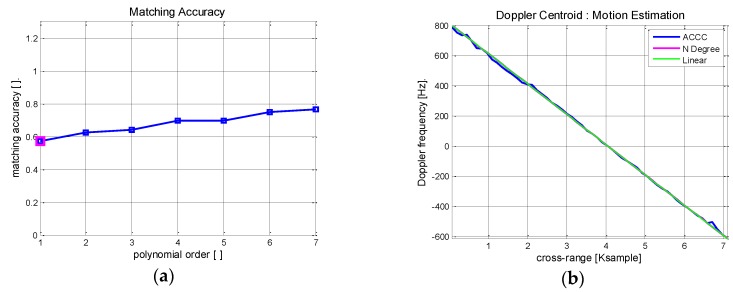
Motion index estimation of Boeing 747 ISAR: (**a**) Matching accuracy of polynomial fitting; (**b**) Doppler polynomial fitting results.

Now, MOCO process is implemented using the estimated Doppler parameters along the range trajectory. The best fitting trajectory model is explored by searching for the acceptable degree of the matching polynomial. The procedure is illustrated in Figure 13.

The coefficients of the approximating polynomial are evaluated that best match the previously obtained Doppler history. The matching accuracy is defined as the ratio of the inlier samples to the total azimuth points. Figure 13a shows the calculated matching accuracy of the Boeing 747 ISAR data for the given polynomial order or motion index. The red marked point corresponds to matching accuracy of 0.6 and it is increased up to 0.8 for *N* = 7. The choice of acceptable motion index is a function of the target stability. 

Figure 13b shows the previously estimated Doppler history (blue) and the polynomial fitting curve (red) of *N* = 2. For comparison, a linear approximation curve is plotted as a straight line (green). The polynomial fitting curve is mostly overlaid by the green linear function. Higher motion index is beneficial for the accurate estimation but it comes with increased computational burden. In this experiment, it is found that the motion index of *N* = 2 provides a good estimation of the Doppler history. Finally, the range and phase errors are calculated using Equation (15) from the generated polynomial fitting model and corrected by compensation.

Once the Doppler history based MOCO is completed, the full aperture processing can be initiated. It is noted that the Doppler bandwidth from Boeing 747 signal is 1453 Hz as compared to the system PRF of 800 Hz. In this case, the use of the range-Doppler algorithm is not suitable as it would incur aliasing problem. To cope with this problem, the proposed ISAR-to-SAR approach scheme is employed to make use of the full aperture data without increasing the system PRF.

**Figure 14:**
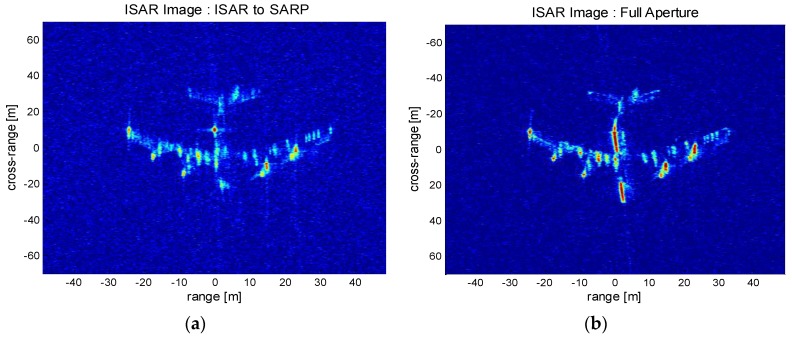
Processed high resolution ISAR image (Boeing 747): (**a**) Sliced aperture data limited by PRF aperture; (**b**) Full aperture data.

For demonstration, Figure 14 compares the distinguished performances between the limited and fully expanded apertures. The time sliced ISAR image taken from conventional range-Doppler processing is presented in Figure 14a. The processed aperture length is limited by the PRF time to avoid azimuth aliasing. The generated ISAR image presents the coarse layout of the aircraft but the detailed features are either missing or not clearly visible. On the other hand, the ISAR-to-SAR processing generates a fully processed ISAR image as shown in Figure 14b. It is seen that the detailed aircraft feature is well preserved, which is attributed to the enhanced SNR and high resolution properties of the full aperture processing. The aircraft body is clearly observed, while the engine structures along the wings are detected with sharp contrast. The clear distinction between the constructed ISAR images validates the superior performance of the full aperture algorithm over the limited aperture case. It can be inferred that the unique advantage of the proposed algorithm would be highly useful for target recognition and classification.

### 5.2. Processing Results of Cessna 208

The Doppler history of a fast-moving target converges toward a simple linear function. On the other hand, a small target flying at relatively low speed is easily disturbed and becomes vulnerable to the non-linear Doppler regression. In that case, the difficulty of Doppler history estimation is escalated in proportion to the aperture lengths. In this section, the previous work is repeated against Cessna 208 that flies at relatively low speed. The small sized aircraft is more vulnerable to the atmospheric disturbance and thus the non-linear Doppler shift effect is intensified.

**Figure 15:**
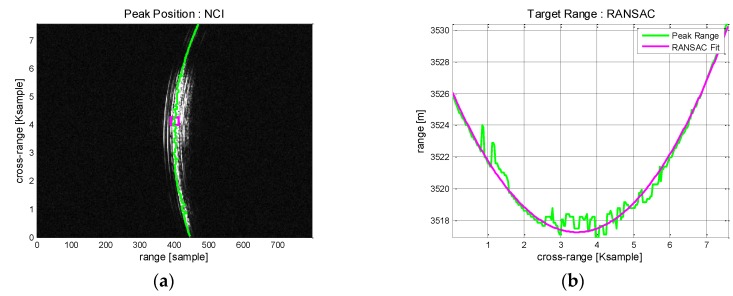
Cessna 208 ISAR data after range compression: (**a**) Range compressed pattern; (**b**) Range tracking result.

Figure 15 shows the range compressed pattern and range tracking result obtained from the echo signal of Cessna 208. Compared with the Boeing aircraft, the prominent scatterers are less detected and the range curvatures are relatively low.

**Figure 16:**
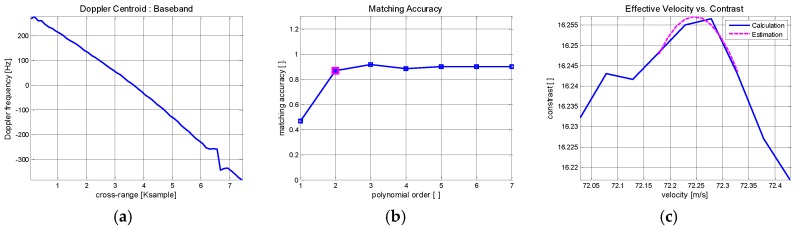
Doppler history estimation of Cessna 208 ISAR data: (**a**) ACCC based Doppler history estimation results; (**b**) Motion index estimation; (**c**) Effective velocity estimation.

By applying the same procedure as depicted in previous section, Doppler parameter estimation is carried out and the results are shown in Figure 16. ACCC method is applied to estimate instantaneous Doppler frequency in Figure 16a and the Doppler bandwidth is measured as 690 Hz. Unlike Boeing 747, this is lower than the system PRF of 800 Hz and thus phase unwrapping is not necessary. The motion index is evaluated in Figure 16b. The matching accuracy becomes saturated at the second order polynomial and it is chosen as the minimum motion index. Effective velocity is estimated via CMA and obtained as 72.24 m/s in Figure 16c.

**Figure 17:**
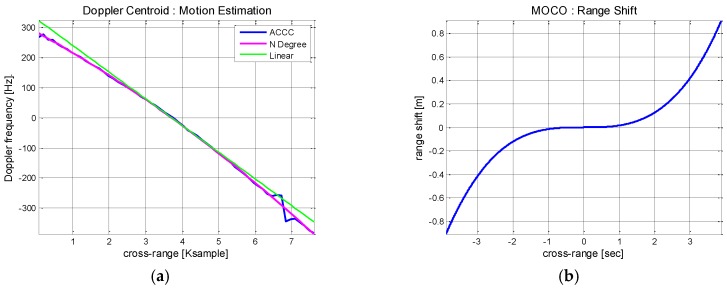
Range error estimation based Doppler history analysis: (**a**) Polynomial fitting results; (**b**) Range shift error calculation.

The range errors are measured from the estimated Doppler history through polynomial fitting. The polynomial fitting graph is compared with the linear regression along with ACCC result in Figure 17a.

As predicted from Figure 16, the estimated Doppler curve deviates from the linear trajectory. The range shift errors are measured from the estimated Doppler history and shown in Figure 17b. It is noted that the non-linear motion has resulted in enlarged range errors. While the complexity of accurate MOCO has further intensified, the Doppler history based correction is still valid and adopted to compensate for the range shifting errors.

**Figure 18:**
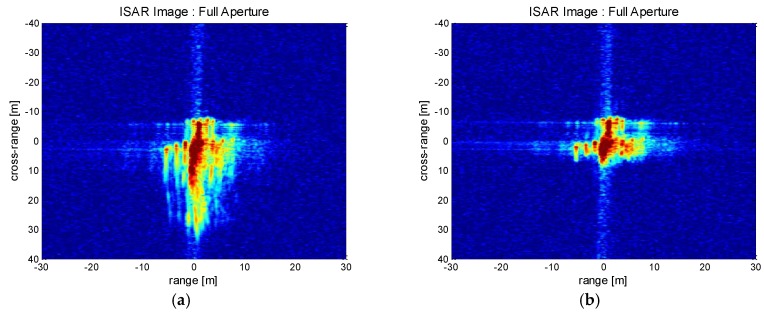
Processed high resolution ISAR image (Cessna 208): (**a**) Initial ISAR processing result; (**b**) After Doppler history based MOCO.

Figure 18 shows the generated ISAR images produced by the ISAR-to-SAR full aperture processing. Initially the defocusing error results in severe blurring and the target recognition is not straightforward (Figure 18a). Afterward, the MOCO algorithm based on Doppler history correction is applied to generate Figure 18b. Now, the blurring effect is mostly removed and the details of the aircraft feature is recognised. Therefore, it is claimed that the proposed method of using Doppler based estimation and correction is advantageous in dealing with the wide aperture ISAR data. The proposed ISAR imaging technique is robustly applicable for the classification of the disturbed targets in remote distance.

## 6. Conclusions

In this paper, a novel high resolution ISAR processing technique is proposed to take full advantage of wide aperture data. The proposed method is based on the ISAR-to-SAR approach that makes use of high resolution spotlight SAR mode and the sub-aperture recombination scheme.

We have demonstrated that the Doppler history of the received ISAR signal can be accurately traced and retrieved with the best fitting model likewise spotlight SAR processing. The proposed algorithm is dedicated to ISAR target having wide aperture interval and exhibits robust MOCO performance against unstable targets having non-linear motions.

When the ISAR aperture interval is extended, conventional range-Doppler algorithm is not a suitable choice due to the intensified difficulty of resolving the escalated range errors and motion corruptions. In this paper, it is demonstrated that the combined use of contrast maximization and RANSAC provides sufficiently good computational accuracy and efficiency for the Doppler parameter estimation of the unknown target. While typical ISAR performance is limited by resolution and target motion errors, the proposed technique can efficiently accommodate the wide Doppler bandwidth extension for high resolution imaging. For this purpose, SPECAN algorithm is employed for the sub-aperture processing and recombination to construct spotlight ISAR mode.

The proposed algorithm is verified through fieldwork experiments that have been conducted on aircraft targets flying in remote distances. Despite the simplicity of the ISAR system built with commercial components and no extra calibration scheme, the difficulty of resolving Doppler parameters is well resolved across wide aperture interval. The developed autofocusing strategy is computationally efficient since it does not need any iterative phase compensation as opposed to conventional phase gradient algorithm.

The long observation time of Boeing aircraft target is fully utilized for high resolution ISAR image construction. In addition, the severe motion disturbance of lightweight small aircraft is effectively corrected. Our work will be applicable to high resolution ISAR surveillance for target detection or classification where a rapid and reliable image acquisition is of major concern.

## Figures and Tables

**Table 1:** System specification.

Parameter	Value	Units
Centre frequency	X-band (9.66)	GHz
Pulse width	20	μs
Bandwidth	500	MHz
PRF	<1000	Hz
Tx power	40	dBm
Receiver chain gain	70	dB

**Table 2:** Characteristics of the acquired ISAR data: Boeing 747 and Cessna 208.

Aircraft Model	Target Range	Target Velocity	Exposure Time	Doppler Bandwidth	Elevation Angle
Boeing 747	3.38 km	352 km/h	8.61 s	1453 Hz	≈40°
Cessna 208	3.51 km	259 km/h	7.78 s	690 Hz	≈20°
